# DeSUMOylation of IGF2BP2 Promotes Neuronal Differentiation of OM‐MSCs by Stabilizing SOX11 to Ameliorate Brain Injury After Intracerebral Hemorrhage

**DOI:** 10.1111/cns.70463

**Published:** 2025-06-08

**Authors:** Jun He, Yuhan Luo, Chuang Wang, Chonghua Jiang, Jian Wang, Ying Xia

**Affiliations:** ^1^ Department of Neurosurgery Haikou Affiliated Hospital of Central South University Xiangya School of Medicine Haikou Hainan Province P. R. China; ^2^ The Second People's Hospital of Changde Changde Hunan Province P. R. China

**Keywords:** ICH, IGF2BP2, neuronal differentiation, OM‐MSCs, SENP1, SOX11

## Abstract

**Background:**

Our previous study demonstrated that olfactory mucosa mesenchymal stem cell (OM‐MSC) neuronal differentiation can reduce neural damage following intracerebral hemorrhage (ICH). However, the mechanisms that regulate OM‐MSC neuronal differentiation to mitigate ICH‐induced brain injury remain unclear.

**Methods:**

The ICH model was established through autologous blood injection to evaluate the impact of OM‐MSCs on brain injury, using the mNSS method, TUNEL, and Nissl staining, as well as the Western blot assay. qPCR, Western blotting, flow cytometry, and immunofluorescence assays were employed to assess the neuronal differentiation of OM‐MSCs. The SUMOylation assay was conducted to investigate the relationship between IGF2BP2 and SENP1. RIP, RNA pull‐down, and mRNA stability assays were performed to analyze the molecular interaction network involving SENP1, IGF2BP2, and SOX11.

**Results:**

IGF2BP2 enhanced the protective effects of OM‐MSCs against ICH‐induced brain injury, as demonstrated by a significant reduction in brain edema, mNSS scores, and apoptosis, along with improved neuronal survival. Furthermore, the overexpression of IGF2BP2 increased the expression of Tuj‐1, MAP2, NF200, and NeuN, while decreasing GFAP and ALDH1L1 levels, suggesting the stimulatory effects of IGF2BP2 on the neuronal differentiation of OM‐MSCs. Mechanistically, SENP1 enhanced IGF2BP2 expression through SUMO1‐induced IGF2BP2 SUMOylation. Additionally, IGF2BP2 functioned as an RNA‐binding protein for SOX11, thereby increasing SOX11 levels. The depletion of IGF2BP2 negated the SENP1‐induced neuronal differentiation of OM‐MSCs. The overexpression of SOX11 mitigated the inhibitory effects of IGF2BP2 silencing on OM‐MSC neuronal differentiation.

**Conclusion:**

The SENP1/IGF2BP2/SOX11 axis played a crucial role in the neuronal differentiation of OM‐MSCs and ameliorated brain damage caused by ICH.

## Graphical abstract

IGF2BP2 expression is enhanced by SENP1 through the regulation of SUMO1‐induced IGF2BP2 SUMOylation. Additionally, IGF2BP2 promotes the neuronal differentiation of olfactory mucosa‐mesenchymal stem cells (OM‐MSCs) by stabilizing SOX11, which helps to mitigate brain injury following intracerebral hemorrhage.

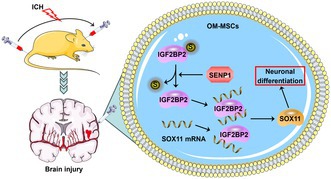

**Abbreviations**
ANOVA: Analysis of variance
FBS: fetal bovine serum
GAPDH: glyceraldehyde 3‐phosphate dehydrogenase
GFAP: glial fibrillary acidic protein
I/R: ischemia/reperfusion
ICH: intracerebral hemorrhage
IGF2: insulin‐like growth factor 2
IGF2BP2: IGF2 mRNA‐binding proteins 2
IP: immunoprecipitation
m6A: N6‐methyladenosine
MAP2: microtubule‐associated protein 2
mNSS: modified neurological severity score
MSCs: Mesenchymal stem cells
NeuN: neuronal nuclei
OM‐MSCs: olfactory mucosa MSCs
PBS: phosphate‐buffered saline
PVDF: polyvinylidene fluoride
qPCR: quantitative real‐time PCR
RBP: RNA‐binding protein
RIPA: radio immunoprecipitation assay
SBI: secondary brain injury
SDS‐PAGE: sodium dodecyl sulfate polyacrylamide gel electrophoresis
SENP1: specific peptidase 1
SOX11: SRY‐box transcription factor 11
STRO‐1: stromal cell antigen 1
SUMO: small ubiquitin‐like modifier
Tuj‐1: beta‐tubulin III
TUNEL: terminal deoxynucleotidyl transferase dUTP nick‐end labeling

## 1 Introduction

Intracerebral hemorrhage (ICH) is a severe cerebrovascular condition characterized by a poor prognosis, high rates of disability, and elevated mortality rates [1, 2]. The unfavorable prognosis of ICH is primarily attributed to the initial brain injury caused by hematoma compression, as well as secondary brain injury (SBI) resulting from the toxic effects of hematoma components [3, 4]. These injuries contribute to neuronal death, ultimately leading to neurological deficits [5]. Numerous studies have examined the effectiveness of surgical interventions to remove hematoma; however, the results are influenced by various factors and remain inconclusive [6]. Effective medical therapies to alleviate SBI are still limited [7]. Therefore, investigating endogenous proteins associated with neuronal differentiation may enhance our understanding of the pathogenesis of ICH and provide new targets for clinical treatment.

Olfactory mucosa MSCs (OM‐MSCs), located in the nasal lamina propria, represent a promising source of stem cells due to their ease of collection and suitability for autologous transplantation [8]. Additionally, OM‐MSCs exhibit multidirectional differentiation capabilities and contribute to the regeneration of the nervous system [9]. Previous reports have revealed that OM‐MSCs play a neuroprotective role in various types of brain injuries. For example, Liu et al. demonstrated that OM‐MSCs relieve cerebral ischemic/reperfusion injury by alleviating mitochondrial dysfunction and enhancing antioxidant activity [10]. Another study indicated that the stereotactic transplantation of OM‐MSCs around the hemorrhagic area reduces neurological deficits caused by ICH [11]. These findings underscore the significant potential of OM‐MSC transplantation for treating brain injuries, including those resulting from ICH. Notably, He et al. unveiled that promoting the differentiation of OM‐MSCs can reduce nerve damage following ICH [12]. However, the underlying mechanisms by which OM‐MSCs modulate neuronal differentiation to alleviate ICH‐induced brain injury require further investigation.

IGF2BP2, a member of the N6‐methyladenosine (m6A) reader proteins, is known to stabilize m6A‐modified mRNAs and enhance their translation [13]. In recent years, additional functional roles of IGF2BP2 in various diseases have been gradually uncovered. For example, as an RNA‐binding protein (RBP), IGF2BP2 regulates osteogenic differentiation in osteoporosis by stabilizing target mRNA [14]. Moreover, Zhu et al. showed that IGF2BP2 plays a role in promoting neurogenesis after hypoxic–ischemic brain injury [15]. Notably, IGF2BP2 is expressed in neocortical neural precursor cells and is involved in regulating the differentiation of these precursor cells into glial cells or neurons [16]. However, it remains unclear whether IGF2BP2, acting as an RBP, regulates ICH‐induced brain damage by regulating the expression of binding RNAs.

The purpose of this study was to investigate the potential of IGF2BP2 in mitigating brain injury induced by ICH by promoting the neuronal differentiation of OM‐MSCs. Specifically, we elucidated the role of the SENP1/IGF2BP2/SOX11 axis in accelerating the neuronal differentiation of OM‐MSCs, thereby providing valuable insights into the therapeutic potential of IGF2BP2 for treating brain injuries caused by ICH.

## 2 Materials and Methods

### 2.1 Animals

All animal experiments were approved by the Animal Care Committee of the Haikou Affiliated Hospital of Central South University Xiangya School of Medicine (ethics approval no. 2024–89). Male C57BL/6 mice, aged 8 to 10 weeks, were obtained from Hunan Slac Jingda Laboratory Animal Co. Ltd. (Changsha, China).

### 2.2 Isolation and Culture of OM‐MSCs

OM‐MSCs were isolated from the olfactory mucosa of C57BL/6 mice. Briefly, the excised nasal mucosal tissue was cultured for 3 days in DMEM/F12 supplemented with 15% fetal bovine serum (FBS) from Gibco Ltd. (USA). OM‐MSCs were expanded until they reached 80% confluence, after which their morphology was observed under an optical microscope. To induce differentiation of OM‐MSCs, the cells were incubated with 1 μM of retinoic acid (Sigma‐Aldrich) and 5 μM of forskolin (Sigma‐Aldrich) for 4 days.

### 2.3 Cell Transfection

For the knockdown of IGF2BP2 or SENP1 expression, shRNAs targeting IGF2BP2 or SENP1 (sh‐IGF2BP2 or sh‐SENP1) and a negative control (sh‐NC) were synthesized and expressed in adenoviral (Ad) vectors (GenePharma). For the overexpression of IGF2BP2, SENP1, or SOX11, pcDNA3.1‐IGF2BP2, SENP1, or SOX11 vectors (oe‐IGF2BP2, oe‐SENP1, or oe‐SOX11) along with oe‐NC expressed in the Ad vectors were obtained from OBiO Technology (Shanghai, China). OM‐MSCs were infected with Ad at an optimal multiplicity of infection of 100.

### 2.4 ICH Model Establishment

The ICH model was established through autologous blood injection [17]. Briefly, C57BL/6 mice were anesthetized with an isoflurane/oxygen mixture, and their body temperature was maintained at 37°C ± 0.5°C using a heated blanket. The mice were then secured in a stereotactic apparatus, and 25 μL of blood from the caudal artery was injected into the caudate nucleus of the right basal ganglia using an injection pump at 2 μL/min. The sham group received an equivalent volume of normal saline.

### 2.5 OM‐MSCs’ Intracerebral Transplantation

The animals were divided into five groups, with six mice per group: sham, ICH, ICH + OM‐MSCs, ICH + OM‐MSCs oe‐NC (OM‐MSCs infected with oe‐NC were injected into mice with ICH), and ICH + OM‐MSCs oe‐IGF2BP2 (OM‐MSCs infected with oe‐IGF2BP2 were injected into mice with ICH). For the OM‐MSC‐triggered groups, 1 × 10^6^ OM‐MSCs in 2 μL of saline were stereotactically injected into the ipsilateral edge of the lesion at 0.1 μL/min, 6 h after the induction of ICH. The ICH group received 2 μL of normal saline in the same location.

### 2.6 Measurement of Cerebral Water Content

The mice were decapitated under deep anesthesia 3 days post‐ICH. The brains were immediately removed and weighed using an analytical microbalance to determine their wet weight. The brain tissues were then dried at 100°C for 24 h and subsequently reweighed to obtain the dry weight. The formula for calculating water content (%) is: (wet weight−dry weight)/wet weight × 100%.

### 2.7 Modified Neurological Severity Score (mNSS)

Using the mNSS, neurological functions—including motor and sensory systems, reflexes, and balance—were assessed on a scale from 0 to 18, with 18 indicating maximum impairment and 0 indicating no impairment.

### 2.8 TUNEL Staining

TUNEL staining was employed to evaluate cell death, adhering to the protocols specified in the In Situ Cell Apoptosis Detection Kit (Roche, USA) manual. In the perihematoma region, TUNEL‐positive cells were captured using a fluorescence microscope and analyzed with ImageJ software (ImageJ 1.5, NIH, USA).

### 2.9 Nissl Staining

After routine dewaxing, brain sections were immersed in a Nissl staining solution (C0117, Beyotime, Shanghai, China) and stained at 37°C for 6 min. The sections were rinsed with deionized water, followed by dehydration and clarification. Subsequently, the sections were sealed overnight with neutral resin and examined under an inverted microscope (Olympus, Tokyo, Japan). The surviving neurons were quantified using ImageJ software.

### 2.10 Flow Cytometry

To identify OM‐MSCs, isolated OM‐MSCs were stained with anti‐CD34 (ab81289, Abcam), anti‐CD45 (ab10558, Abcam), anti‐CD73 (ab288154, Abcam), anti‐CD105 (ab221675, Abcam), anti‐CD44 (ab243894, Abcam), and anti‐CD90 (ab307736, Abcam). To detect Tuj‐1‐positive cells, the cells were digested with trypsin and re‐suspended in phosphate‐buffered saline (PBS) containing Triton X‐100 and 1 mL of 4% paraformaldehyde. Subsequently, the cells were blocked with sheep serum and incubated with an antibody against Tuj‐1 (ab18207, Abcam). After incubation with the appropriate secondary antibodies, the cells were analyzed using a FACSCalibur and FlowJo software.

### 2.11 Immunofluorescence

The prepared OM‐MSCs or tissues were fixed in 4% paraformaldehyde for 15 min, permeabilized with 0.3% Triton X‐100 for 10 min, and blocked with 5% bovine serum albumin for 1 h. Subsequently, they were incubated with primary antibodies, including Nestin (ab316018,1/250, Abcam), STRO‐1 (39–8401, 1/200, Invitrogen), GFAP (ab7260, 1/5000, Abcam), and NeuN (ab177487, 1/100, Abcam). Following washing with PBS, the tissues or cells were incubated in the dark with the appropriate fluorescence‐conjugated secondary antibodies for 1 h, followed by DAPI staining. Immunofluorescence images were captured with a fluorescence microscope (Leica). The target‐positive cells were quantified by a blinded investigator using NIH ImageJ software. Three randomly selected microscopic fields from each section were analyzed, and the immune‐positive cells were reported as the mean percentage of cells per field.

### 2.12 qPCR Assay

Total RNA was extracted using TRIzol reagent and reverse transcribed into cDNA using the PrimeScript RT reagent kit (Takara, Japan). Subsequently, qPCR was conducted using SYBR Green Master Mix on an ABI 7500 real‐time PCR system. GAPDH mRNA served as the control, and the 2^−ΔΔ*C*^ ^t^ method was utilized to quantify gene expression. The primers are listed in Table 1.

**TABLE 1:** Sequences of primers for qPCR analysis.

Gene	Primer sequence
IGF2BP2	Forward: 5′‐TGAAGCCTGTGCCAATGCTGAG‐3′
	Reverse: 5′‐CCAGTCGAAAAGATGCCAAGTGC‐3′
Tuj‐1	Forward: 5′‐CATCAGCGATGAGCACGGCATA‐3′
	Reverse: 5′‐GGTTCCAAGTCCACCAGAATGG‐3′
GFAP	Forward: 5′‐CACCTACAGGAAATTGCTGGAGG‐3′
	Reverse: 5′‐CCACGATGTTCCTCTTGAGGTG‐3′
MAP2	Forward: 5′‐GCTGTAGCAGTCCTGAAAGGTG‐3′
	Reverse: 5′‐CTTCCTCCACTGTGGCTGTTTG‐3′
SOX11	Forward: 5′‐GGACCTGGATTCCTTCAGTGAG‐3′
	Reverse: 5′‐GTGAACACCAGGTCGGAGAAGT‐3′
GAPDH	Forward: 5′‐CATCACTGCCACCCAGAAGACTG‐3′
	Reverse: 5′‐ATGCCAGTGAGCTTCCCGTTCAG‐3′
NF200	Forward: 5′‐TAGAGGACCGTCATCAGGCAGA‐3′
	Reverse: 5′‐TGACGTTGAGCAGGTCCTGGTA‐3′
ALDH1L1	Forward: 5′‐CTTCATAGGCGGCGAGTTTGTG‐3′
	Reverse: 5′‐CGCCTTGTCAACATCACTCACC‐3′

### 2.13 Western Blotting

Total protein was extracted using RIPA buffer (Beyotime). After quantification with a BCA Kit (Beyotime), equal amounts of protein were separated by SDS‐PAGE, transferred onto PVDF membranes, and blocked with 5% non‐fat milk. The membranes were incubated overnight at 4°C with primary antibodies: Bax (ab32503, 1/2000, Abcam), Bcl‐2 (ab182858, 1/2000, Abcam), cleaved Caspase3 (ab214430, 1/5000, Abcam), IGF2BP2 (ab128175, 1/1000, Abcam), SENP1 (ab236094, 1/500, Abcam), SOX11 (ab234996, 1/500, Abcam), SUMO1 (ab133352, 1/1000, Abcam), and β‐actin (ab8227, 1/2000, Abcam), followed by HRP‐conjugated secondary antibodies. Protein bands were visualized using an ECL Detection Kit (Invitrogen), quantified with ImageJ software, and normalized to β‐actin as a loading control.

### 2.14 Immunoprecipitation (IP) Assay for Measurement of SUMOylation

Treated OM‐MSCs were washed three times with cold PBS and lysed in IP buffer. The cell lysates were centrifuged, and the supernatants were precipitated using either the IGF2BP2 antibody (ab128175, Abcam) or the SUMO1 antibody (ab32058, Abcam), along with an IgG antibody. The cells were incubated overnight at 4°C with Protein A/G agarose beads. The washed precipitated proteins were analyzed by Western blotting using SUMO1 and IGF2BP2 antibodies.

### 2.15 RNA IP (RIP) Assay

Cells were lysed on ice using RIP lysis buffer (Millipore) for 30 min. The cell extracts were then incubated with RIP buffer containing magnetic beads conjugated to antibodies against IgG (ab172730, Abcam) or IGF2BP2 (ab128175, Abcam) for 2 h. After washing, the magnetic beads were re‐suspended in TRIzol, and the co‐precipitated RNAs were isolated to detect SOX11 mRNA levels using a qPCR assay.

### 2.16 RNA Pull‐Down Assay

A biotin‐labeled full‐length SOX11 RNA probe was prepared using the Biotin RNA Labeling Mix (Roche, Basel, Switzerland). OM‐MSCs, with a minimum of 1 × 10^7^ cells, were collected and lysed. The cell lysates were then mixed with biotin‐SOX11 probes at 4°C for 1 h and subsequently incubated with Pierce Streptavidin Magnetic Beads (Thermo Scientific, USA) for 2 h. After extensive washing, the RNA‐protein complexes were eluted and subjected to Western blot analysis to detect IGF2BP2 binding.

### 2.17 mRNA Stability Assay

After treatment, OM‐MSCs were incubated with 5 μg/mL of actinomycin D for 0, 3, and 6 h. Subsequently, RNA was extracted from the cells, reverse transcribed into cDNA, and analyzed for the remaining SOX11 mRNA using qPCR.

### 2.18 Statistical Analysis

All statistical analyses were conducted using GraphPad Prism 8 (GraphPad Software, San Diego, CA, USA). Data are presented as the mean ± standard deviation (mean ± SD) from a minimum of three independent experiments. Normality was assessed using the Shapiro–Wilk test, and all data were found to be normally distributed in this study. Group differences were evaluated using either Student's *t*‐test or one‐way ANOVA, followed by Tukey's post hoc test. A *p*‐value of less than 0.05 was considered statistically significant for all tests.

## 3 Results

### 3.1 Identification of the Isolated OM‐MSCs

**FIGURE 1:**
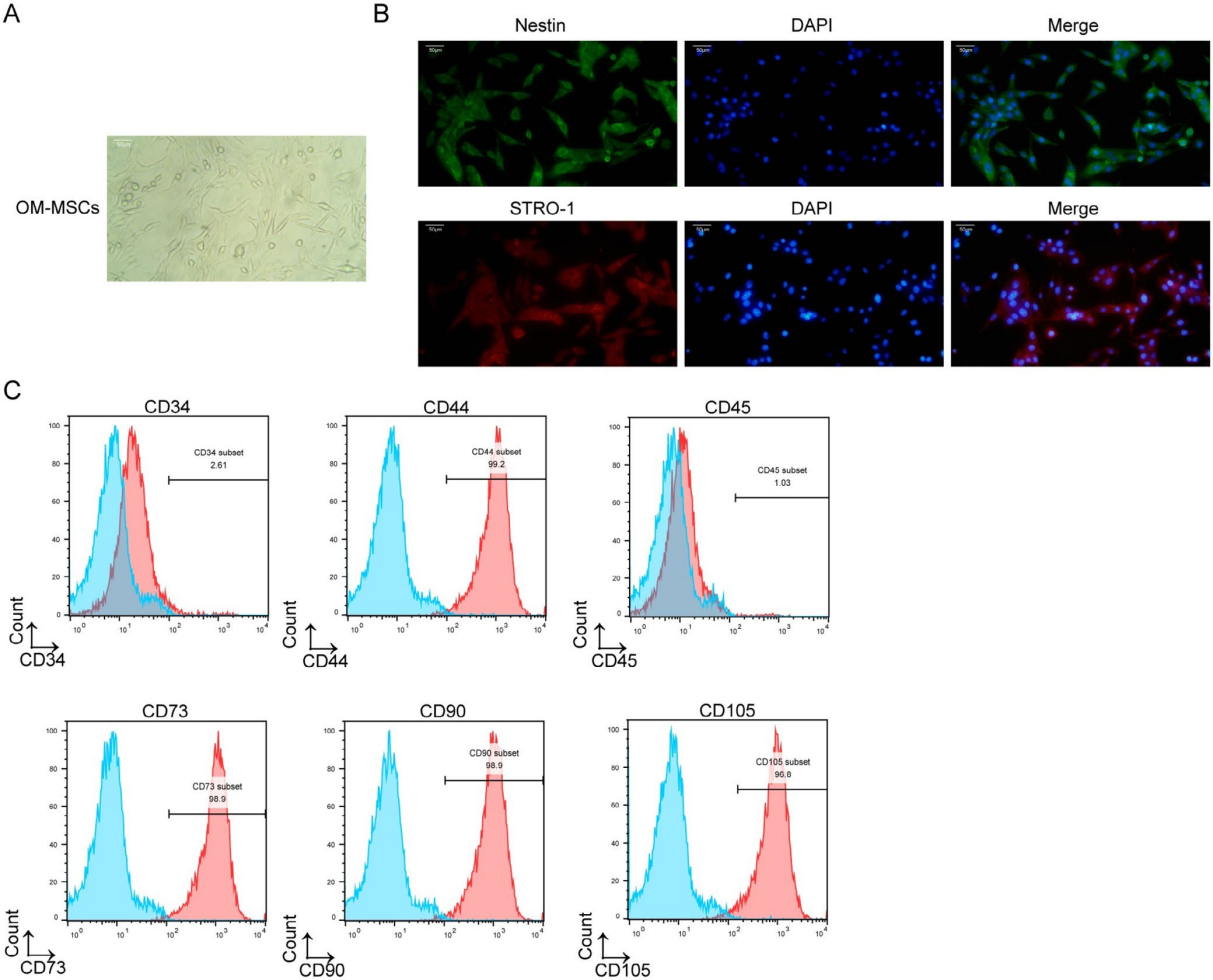
Identification of the isolated OM‐MSCs. (A) OM‐MSCs mainly exhibited spindle‐shaped and a radial arrangement under a light microscope. Scale bar, 50 μm. (B) Specific markers STRO‐1 and Nestin of OM‐MSCs were identified by immunofluorescence. Scale bar, 50 μm. (C) Surface markers (CD34, CD44, CD45, CD73, CD90, and CD105) of OM‐MSCs were measured by flow cytometry assay. *n* = 3.

Under a light microscope, isolated cells exhibited a spindle‐shaped morphology (Figure 1A). Additionally, the purified cells tested positive for Nestin and STRO‐1, which are characteristic markers of OM‐MSCs (Figure 1B). The presence of cell surface markers was further confirmed through flow cytometry analysis, revealing that the obtained cells expressed positive markers (CD44, CD73, CD90, and CD105) and negative markers (CD45 and CD34) (Figure 1C). These results indicate the successful isolation of OM‐MSCs.

### 3.2 IGF2BP2 Expedited the Protective Effect of OM‐MSCs Against ICH‐Caused Brain Injury

**FIGURE 2:**
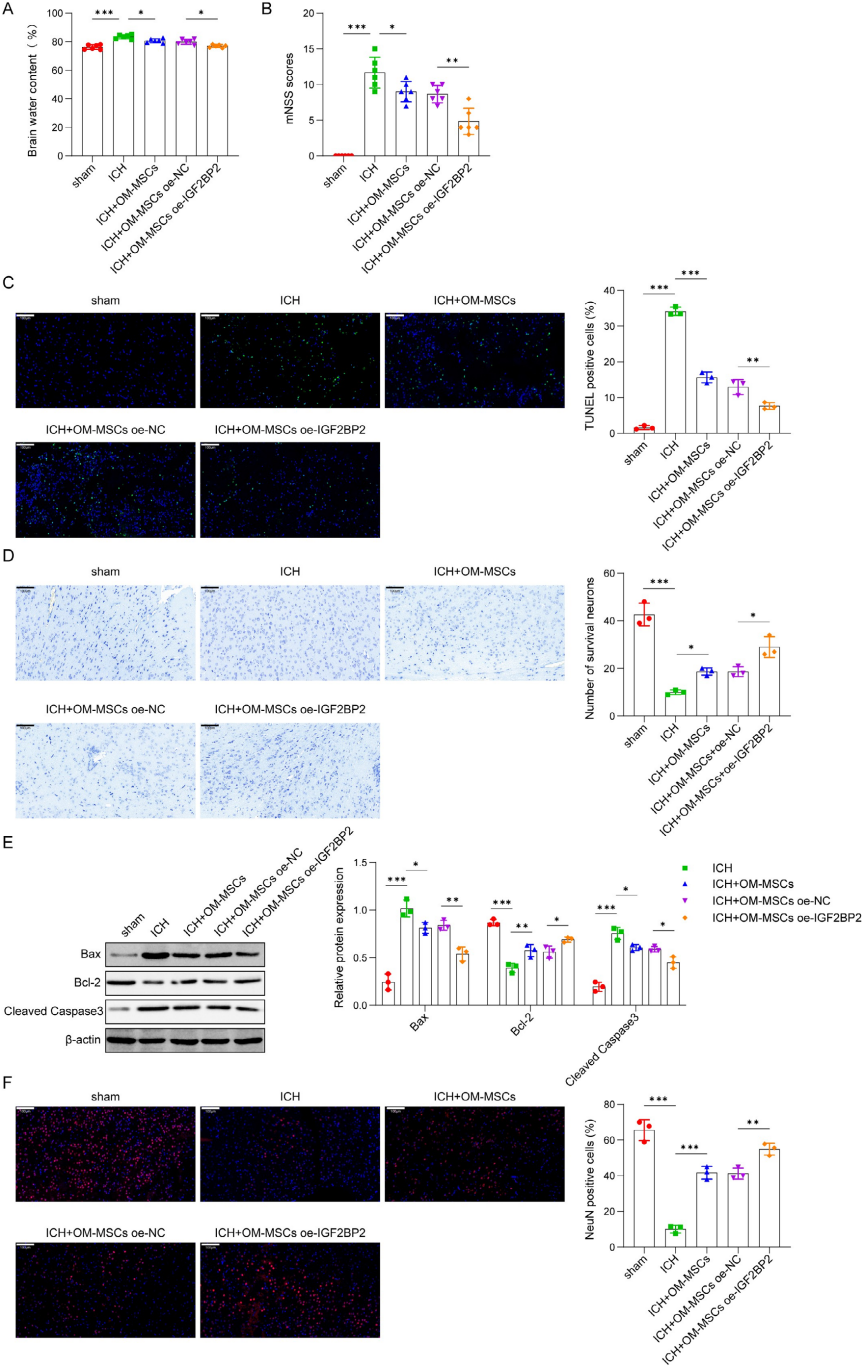
IGF2BP2 expedited the protective effect of OM‐MSCs against ICH‐caused brain injury. (A) Analysis of brain water content in brain region at 72 h after ICH, which was performed in mice in sham (76.25 ± 1.66), ICH (83.48 ± 1.49), ICH + OM‐MSCs (80.63 ± 1.42), ICH + OM‐MSCs oe‐NC (79.93 ± 1.71), and ICH + OM‐MSCs oe‐IGF2BP2 (76.92 ± 1.05) groups. *n* = 6. (B) mNSS scoring (ICH: 11.67 ± 2.16, ICH + OM‐MSCs: 9.00 ± 1.41, ICH + OM‐MSCs oe‐NC: 8.67 ± 1.12, ICH + OM‐MSCs oe‐IGF2BP2: 4.83 ± 1.84) of mice after 72 h of ICH modeling. *n* = 6. (C) Representative images of TUNEL in the perihematoma area of mice after 72 h of ICH (scale bar, 100 μm) and TUNEL positive cells (sham: 1.62 ± 0.55, ICH: 34.15 ± 1.16, ICH + OM‐MSCs: 15.63 ± 1.49, ICH + OM‐MSCs oe‐NC: 12.97 ± 2.09, ICH + OM‐MSCs oe‐IGF2BP2: 7.65 ± 0.93) were counted. (D) Nissl staining was conducted in the perihematoma area of mice after 72 h of ICH (cale bar, 100 μm) and the number of survival neurons (sham: 42.67 ± 4.73, ICH: 10.00 ± 1.00, ICH + OM‐MSCs: 18.67 ± 1.53, ICH + OM‐MSCs oe‐NC: 18.67 ± 2.08, ICH + OM‐MSCs oe‐IGF2BP2: 29.00 ± 4.36) was counted. (E) Bax (sham: 0.25 ± 0.08, ICH: 1.02 ± 0.09, ICH + OM‐MSCs: 0.81 ± 0.06, ICH + OM‐MSCs oe‐NC: 0.84 ± 0.05, ICH + OM‐MSCs oe‐IGF2BP2: 0.54 ± 0.07), cleaved Caspase3 (sham: 0.20 ± 0.05, ICH: 0.76 ± 0.06, ICH + OM‐MSCs: 0.61 ± 0.03, ICH + OM‐MSCs oe‐NC: 0.59 ± 0.03, ICH + OM‐MSCs oe‐IGF2BP2: 0.45 ± 0.06), and Bcl‐2 (sham: 0.87 ± 0.03, ICH: 0.39 ± 0.05, ICH + OM‐MSCs: 0.57 ± 0.06, ICH + OM‐MSCs oe‐NC: 0.56 ± 0.06, ICH + OM‐MSCs oe‐IGF2BP2: 0.69 ± 0.03) protein levels were measured by western blot assay in the above groups. (F) Immunofluorescence was used to observe the number of NeuN‐positive cells (sham: 65.54 ± 5.84, ICH: 10.11 ± 2.16, ICH + OM‐MSCs: 41.70 ± 3.53, ICH + OM‐MSCs oe‐NC: 41.23 ± 3.12, ICH + OM‐MSCs oe‐IGF2BP2: 54.90 ± 3.32) in the perihematoma area of mice after 72 h of ICH. Scale bar, 100 μm. *n* = 3. *p < 0.05, **p < 0.01, ***p < 0.001.

Previous research has demonstrated the protective effect of OM‐MSCs against brain injury induced by ICH [11, 18]. In this study, we evaluated whether IGF2BP2 is involved in the protective effect of OM‐MSCs against ICH‐induced brain injury. As illustrated in Figure 2A,B, at 72 h post‐ICH, mice exhibited significantly increased brain water content and elevated mNSS, both of which were effectively mitigated by OM‐MSCs treatment. Interestingly, the overexpression of IGF2BP2 further enhanced the effects of OM‐MSCs on brain water content and mNSS score. Moreover, apoptosis (Figure 2C) increased and neuronal survival (Figure 2D) decreased in mice after ICH, but these effects were mitigated by OM‐MSCs administration. The overexpression of IGF2BP2 further enhanced the impact of OM‐MSCs on reducing apoptosis and promoting neuronal survival. Besides, in ICH mice, Bax and cleaved Caspase3 protein levels enhanced while Bcl‐2 protein levels decreased; however, these changes were reversed by OM‐MSCs, with IGF2BP2 overexpression further amplifying the effect of OM‐MSCs (Figure 2E). Further, the neuronal marker NeuN was reduced in ICH mice; however, this change was partially reversed by the administration of OM‐MSCs. Additionally, IGF2BP2 further enhanced the increase in NeuN‐positive staining (Figure 2F). Taken together, IGF2BP2 may enhance the therapeutic effects of OM‐MSCs on ICH‐caused brain injury, and may also play a role in the neuronal differentiation of OM‐MSCs.

### 3.3 IGF2BP2 Facilitated Neuronal Differentiation of OM‐MSCs

**FIGURE 3:**
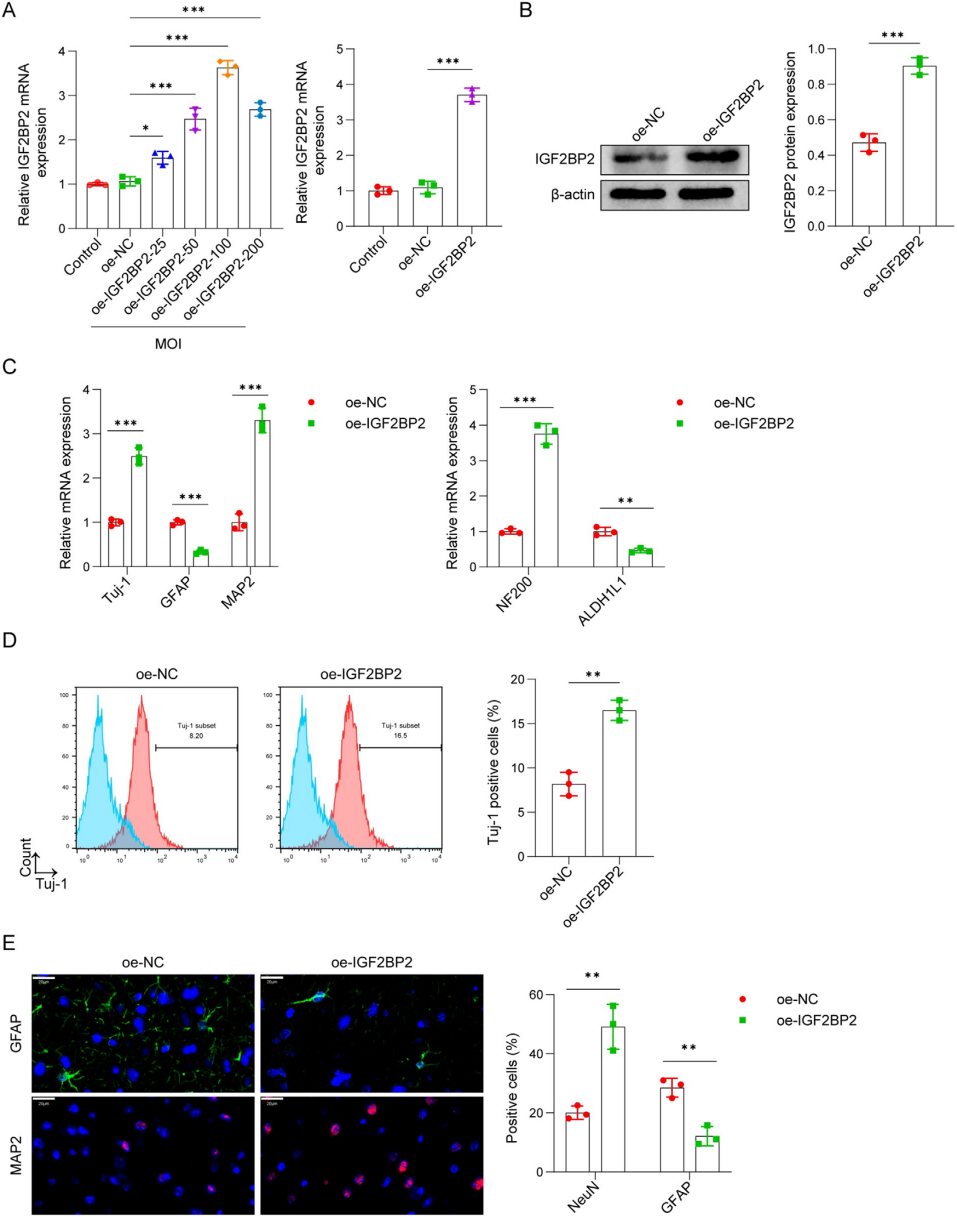
IGF2BP2 facilitated neuronal differentiation of OM‐MSCs. (A) OM‐MSCs were infected with adenoviral vectors (oe‐IGF2BP2) at the MOIs of 25–200 (control: 1.00 ± 0.04, oe‐NC: 1.07 ± 0.10, oe‐IGF2BP2–25: 1.59 ± 0.14, oe‐IGF2BP2–50: 2.47 ± 0.25, oe‐IGF2BP2–100: 3.63 ± 0.16, oe‐IGF2BP2–200: 2.69 ± 0.15) for 48 h and the mRNA expression of IGF2BP2 was detected using qPCR in control (1.00 ± 0.11), oe‐NC (1.09 ± 0.18), and oe‐IGF2BP2 (3.70 ± 0.19) groups. (B) OM‐MSCs were infected with adenoviral vectors at an optimal multiplicity of 100 for 48 h and IGF2BP2 protein levels were measured by western bolt assays in oe‐NC (0.47 ± 0.05) and oe‐IGF2BP2 (0.90 ± 0.05) groups. (C) OM‐MSCs were infected with Ad and incubated with differentiation medium for four days. The mRNA levels of Tuj‐1 (oe‐NC: 1.00 ± 0.08, oe‐IGF2BP2: 2.50 ± 0.18), MAP2 (oe‐NC: 1.00 ± 0.18, oe‐IGF2BP2: 3.31 ± 0.28), NF200 (oe‐NC: 1.00 ± 0.07, oe‐IGF2BP2: 3.75 ± 0.29), GFAP (oe‐NC: 1.00 ± 0.06, oe‐IGF2BP2: 0.33 ± 0.05), and ALDH1L1 (oe‐NC: 1.00 ± 0.12, oe‐IGF2BP2: 0.46 ± 0.06) was measured by qPCR assay. (D) Flow cytometry assay was used to detect Tuj‐1 positive cells (oe‐NC: 8.19 ± 1.33, oe‐IGF2BP2: 16.50 ± 1.15). (E) Immunofluorescence was used to observe the number of GFAP (oe‐NC: 28.55 ± 3.16, oe‐IGF2BP2: 12.14 ± 3.26) and NeuN (oe‐NC: 20.07 ± 2.27, oe‐IGF2BP2: 49.14 ± 7.57)‐positive cells. Scale bar, 20 μm. *n* = 3. *p < 0.05, **p < 0.01, ***p < 0.001.

As shown in Figure 3A, IGF2BP2 mRNA expression increased in a dose‐dependent manner across multiplicities of infection (MOIs) of 25, 50, and 100, with an MOI of 100 selected for further experiments. These results confirm the successful establishment of a dose gradient for IGF2BP2 expression. We overexpressed IGF2BP2 in OM‐MSCs and evaluated IGF2BP2 expression in Figure 3A,B. Addition of IGF2BP2 distinctly enhanced IGF2BP2 mRNA and protein levels. To probe the effect of IGF2BP2 on neuronal differentiation, we assessed neuronal markers (Tuj‐1, MAP2, and NF200) and glial markers (GFAP and ALDH1L1) in OM‐MSCs. qPCR analysis revealed that IGF2BP2 overexpression significantly upregulated neuronal markers (Tuj‐1, MAP2, and NF200) while downregulating glial markers (GFAP and ALDH1L1) (Figure 3C). Moreover, flow cytometry (Figure 3D) and immunofluorescence (Figure 3E) assays confirmed that overexpression of IGF2BP2 enhanced Tuj‐1 and NeuN‐positive cells while decreasing GFAP‐positive cells. These results confirm the stimulating influence of IGF2BP2 on OM‐MSC neuronal differentiation.

### 3.4 SENP1 Regulated the SUMOylation of IGF2BP2

**FIGURE 4:**
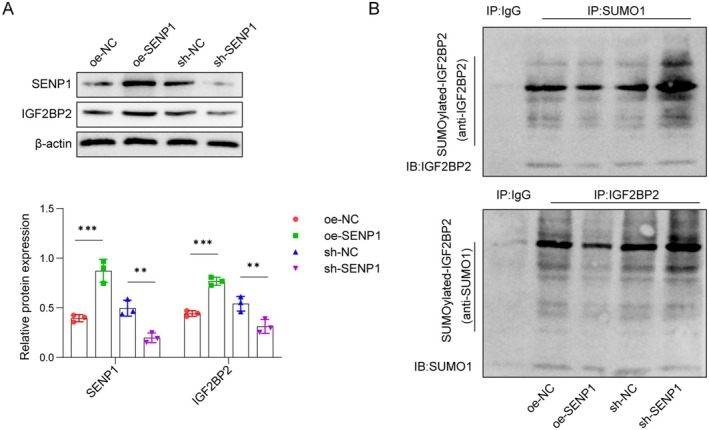
SENP1 regulated the SUMOylation of IGF2BP2. (A) OM‐MSCs were infected with adenoviral vectors at an optimal multiplicity of 100 for 48 h. Western blot analysis of SENP1 (oe‐NC: 0.39 ± 0.04, oe‐SENP1: 0.87 ± 0.11, sh‐NC: 0.50 ± 0.08, sh‐SENP1: 0.20 ± 0.05) and IGF2BP2 (oe‐NC: 0.44 ± 0.03, oe‐SENP1: 0.77 ± 0.04, sh‐NC: 0.54 ± 0.07, sh‐SENP1: 0.31 ± 0.07) protein levels in OM‐MSCs treated with oe‐NC, oe‐SENP1, sh‐NC, or sh‐SENP1. (B) OM‐MSCs were infected with adenoviral vectors and IGF2BP2 SUMOylation was assessed by immunoprecipitation/western blot. Cell lysates were collected and subjected to immunoprecipitation using anti‐SUMO1 or anti‐IGF2BP2. Immunoblot analysis was then performed using the specified antibodies. *n* = 3. **p < 0.01, ***p < 0.001.

As illustrated in Figure 4A, SENP1 addition enhanced, while SENP1 depletion decreased, the protein levels of both SENP1 and IGF2BP2. Subsequent IP experiments confirmed the interaction between SUMO1 and IGF2BP2, which is regulated by SENP1. SENP1 decreased the SUMO1‐induced SUMOylation of IGF2BP2, whereas silencing SENP1 resulted in the opposite effect (Figure 4B). These findings indicate that SENP1 modulates the neuronal differentiation of OM‐MSCs by controlling SUMO1‐induced IGF2BP2 SUMOylation.

### 3.5 IGF2BP2 Was Implicated in SENP1‐Mediated OM‐MSC Neuronal Differentiation

**FIGURE 5:**
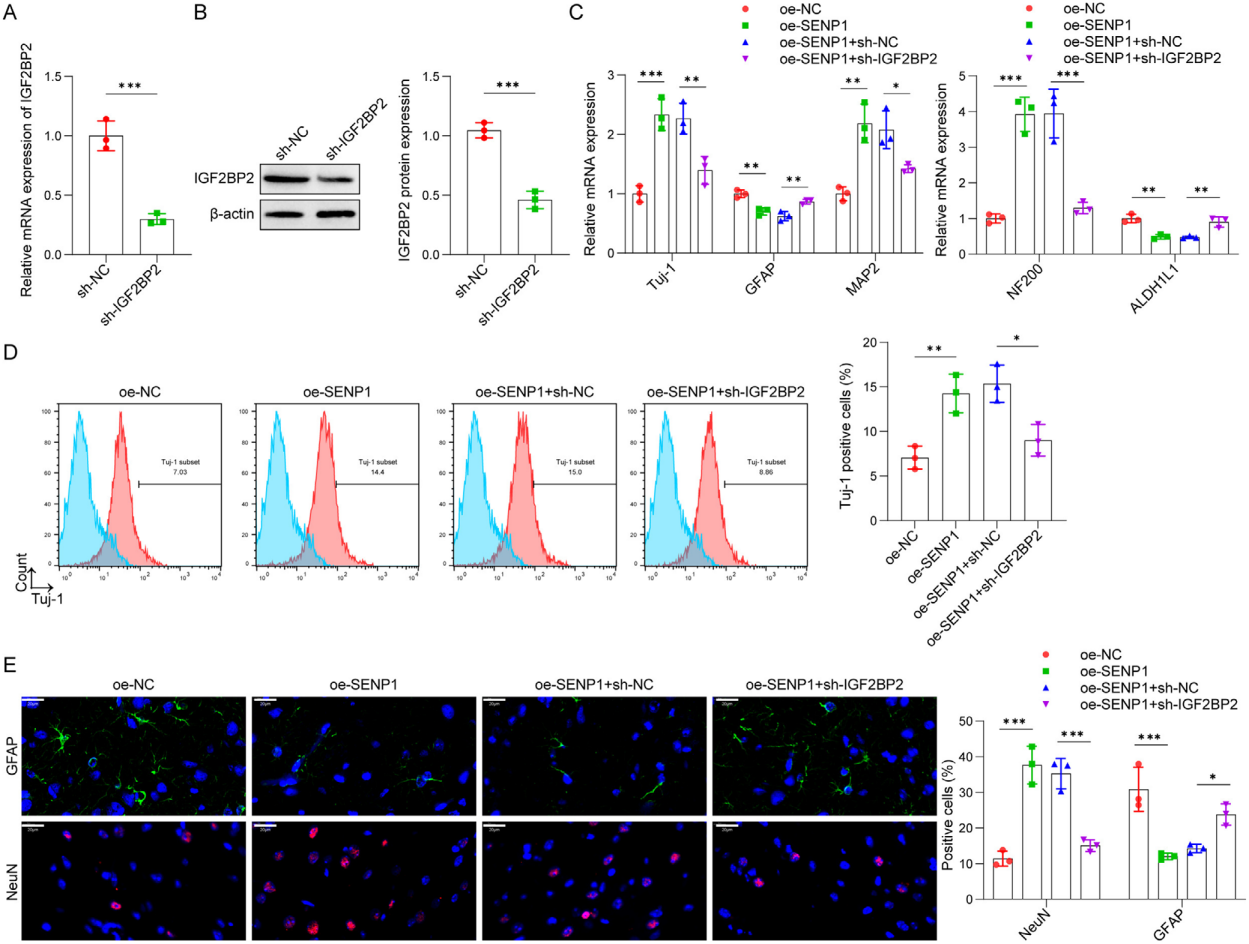
IGF2BP2 was implicated in SENP1‐mediated OM‐MSC neuronal differentiation. (A and B) qPCR (sh‐NC: 1.00 ± 0.13, sh‐IGF2BP2: 0.30 ± 0.05) and western blot (sh‐NC: 1.05 ± 0.06, sh‐IGF2BP2: 0.46 ± 0.07) analysis of IGF2BP2 mRNA and protein levels in OM‐MSCs treated with sh‐NC or sh‐IGF2BP2 for 48 h. (C) OM‐MSCs were infected with Ad and incubated with differentiation medium for four days. The mRNA levels of Tuj‐1 (oe‐NC: 1.00 ± 0.14, oe‐SENP1: 2.33 ± 0.27, oe‐SENP1 + sh‐NC: 2.27 ± 0.26, oe‐SENP1 + sh‐IGF2BP1: 1.40 ± 0.23), MAP2 (oe‐NC: 1.00 ± 0.12, oe‐SENP1: 2.19 ± 0.32, oe‐SENP1 + sh‐NC: 2.08 ± 0.32, oe‐SENP1 + sh‐IGF2BP1: 1.43 ± 0.07), NF200 (oe‐NC: 1.00 ± 0.13, oe‐SENP1: 3.92 ± 0.48, oe‐SENP1 + sh‐NC: 3.94 ± 0.69, oe‐SENP1 + sh‐IGF2BP1: 1.30 ± 0.16), GFAP (oe‐NC: 1.00 ± 0.07, oe‐SENP1: 0.70 ± 0.06, oe‐SENP1 + sh‐NC: 0.62 ± 0.08, oe‐SENP1 + sh‐IGF2BP1: 0.87 ± 0.04), and ALDH1L1 (oe‐NC: 1.00 ± 0.12, oe‐SENP1: 0.49 ± 0.07, oe‐SENP1 + sh‐NC: 0.47 ± 0.03, oe‐SENP1 + sh‐IGF2BP1: 0.90 ± 0.14) were measured by qPCR assay. (D) Flow cytometry assay was used to detect Tuj‐1 positive cells (oe‐NC: 7.05 ± 1.29, oe‐SENP1: 14.26 ± 2.16, oe‐SENP1 + sh‐NC: 15.35 ± 2.09, oe‐SENP1 + sh‐IGF2BP1: 9.01 ± 1.77). (E) Immunofluorescence was used to observe the number of GFAP (oe‐NC: 30.84 ± 6.19, oe‐SENP1: 12.04 ± 0.90, oe‐SENP1 + sh‐NC: 14.24 ± 1.17, oe‐SENP1 + sh‐IGF2BP1: 23.79 ± 3.00) and NeuN (oe‐NC: 11.39 ± 2.14, oe‐SENP1: 37.68 ± 5.30, oe‐SENP1 + sh‐NC: 35.24 ± 4.29, oe‐SENP1 + sh‐IGF2BP1: 15.05 ± 1.67)‐positive cells. Scale bar, 20 μm. *n* = 3. *p < 0.05, **p < 0.01, ***p < 0.001.

The knockdown of IGF2BP2 in OM‐MSCs was conducted and the expression levels of IGF2BP2 were measured, as shown in Figure 5A,B. The depletion of IGF2BP2 significantly reduced both mRNA and protein levels of IGF2BP2. To investigate whether IGF2BP2 is involved in SENP1‐mediated OM‐MSC neuronal differentiation, SENP1 was overexpressed in OM‐MSCs with or without IGF2BP2 depletion. As illustrated in Figure 5C, SENP1 overexpression upregulated Tuj‐1, MAP2, and NF200 expression, while downregulating GFAP and ALDH1L1 levels; these effects were partially reversed by IGF2BP2 knockdown. Additionally, flow cytometry (Figure 5D) and immunofluorescence (Figure 5E) assays confirmed that overexpression of SENP1 increased Tuj‐1 and NeuN‐positive cells while decreasing GFAP‐positive cells, an effect that was negated by IGF2BP2 silencing. In summary, the enhancement of neuronal differentiation in OM‐MSCs by SENP1 is associated with IGF2BP2.

### 3.6 IGF2BP2 Interacted With SOX11 to Promote Its Expression

**FIGURE 6:**
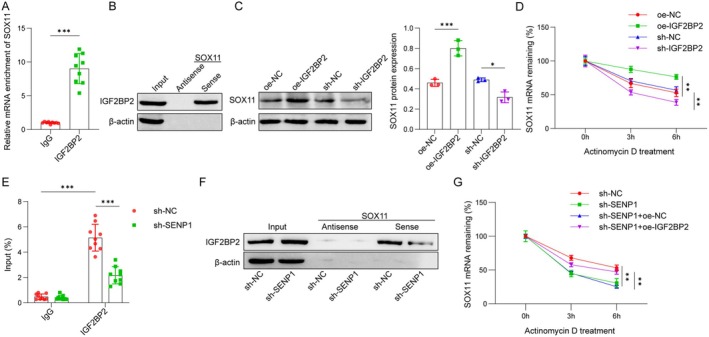
IGF2BP2 interacted with SOX11 to promote its expression. (A and B) RIP (IgG: 1.00 ± 0.13, IGF2BP2: 9.02 ± 2.23) and RNA pull‐down assay verified IGF2BP2 binding to SOX11 mRNA. (C) Western blot analysis of SOX11 protein level in OM‐MSCs treated with oe‐NC (0.46 ± 0.03), oe‐IGF2BP2 (0.80 ± 0.08), sh‐NC (0.49 ± 0.02), or sh‐IGF2BP2 (0.32 ± 0.05). (D) SOX11 mRNA stability in OM‐MSCs after oe‐NC (0 h: 100.00 ± 2.32, 3 h: 66.73 ± 6.26, 6 h: 52.90 ± 5.57), oe‐IGF2BP2 (0 h: 100.00 ± 4.61, 3 h: 87.57 ± 4.73, 6 h: 76.45 ± 3.76), sh‐NC (0 h: 100.00 ± 6.74, 3 h: 70.51 ± 3.59, 6 h: 56.78 ± 4.93), or sh‐IGF2BP2 (0 h: 100.00 ± 8.57, 3 h: 53.49 ± 3.88, 6 h: 38.57 ± 4.23) treatment and incubation with actinomycin D for 0, 3, and 6 h was analyzed by qPCR. (E) RIP assay was performed using an anti‐IGF2BP2 antibody in OM‐MSCs with or without SENP1 knockdown. The enrichment of SOX11 mRNA was determined by qPCR (sh‐NC‐IgG: 0.48 ± 0.22, sh‐NC‐IGF2BP2: 5.15 ± 1.06, sh‐SENP1‐IgG: 0.40 ± 0.18, sh‐SENP1‐IGF2BP2: 2.18 ± 0.68). (F) RNA pull‐down assay was conducted using biotin‐labeled SOX11 RNA probes (sense and antisense) in OM‐MSCs transfected with sh‐NC or sh‐SENP1. IGF2BP2 binding was detected by western blot. β‐actin was used as a loading control. (G) OM‐MSCs with indicated treatments (sh‐NC (0 h: 100.00 ± 1.40, 3 h: 67.98 ± 3.76, 6 h: 53.00 ± 4.26), sh‐SENP1 (0 h: 100.00 ± 7.98, 3 h: 44.65 ± 4.82, 6 h: 30.64 ± 6.52), sh‐SENP1 + oe‐NC (0 h: 100.00 ± 3.01, 3 h: 45.24 ± 2.43, 6 h: 25.08 ± 2.08), sh‐SENP1 + oe‐IGF2BP2 (0 h: 100.00 ± 7.90, 3 h: 57.71 ± 2.91, 6 h: 47.08 ± 3.30)) were treated with actinomycin D for 0, 3, and 6 h. SOX11 mRNA levels were measured by qPCR and normalized to the 0‐h time point. *n* = 3. *p < 0.05, **p < 0.01, ***p < 0.001.

Through the ENCORI database (https://rnasysu.com/encori/), we identified IGF2BP2 as the RBP associated with SOX11 mRNA. SOX11 was found to be most abundantly enriched in the precipitates of anti‐IGF2BP2 in OM‐MSCs, indicating an interaction between IGF2BP2 and SOX11 mRNA (Figure 6A,B). Moreover, overexpression of IGF2BP2 increased and depletion of IGF2BP2 decreased SOX11 protein levels as well as SOX11 mRNA stability (Figure 6C,D). As shown in Figure 6E, RIP assay demonstrated that SENP1 knockdown markedly reduced the enrichment of SOX11 mRNA in IGF2BP2‐immunoprecipitated complexes, suggesting that SENP1 promotes the binding between IGF2BP2 and SOX11 mRNA. In Figure 6F, RNA pull‐down assay further confirmed that SOX11 mRNA interacts with IGF2BP2 under control conditions (sh‐NC), while this interaction is weakened upon SENP1 knockdown, indicating that the deSUMOylation activity of SENP1 may be necessary for the proper association of IGF2BP2 with SOX11 mRNA. Additionally, the mRNA stability assay (Figure 6G) revealed that the stability of SOX11 mRNA decreased after SENP1 knockdown, and this effect was reversed by IGF2BP2 overexpression, supporting that SENP1 enhances SOX11 mRNA stability in an IGF2BP2‐dependent manner. Collectively, these results support the specificity of the regulatory interactions among SENP1, IGF2BP2, and SOX11 in modulating SOX11 mRNA stability.

### 3.7 IGF2BP2 Facilitated OM‐MSC Neuronal Differentiation by Increasing SOX11

**FIGURE 7:**
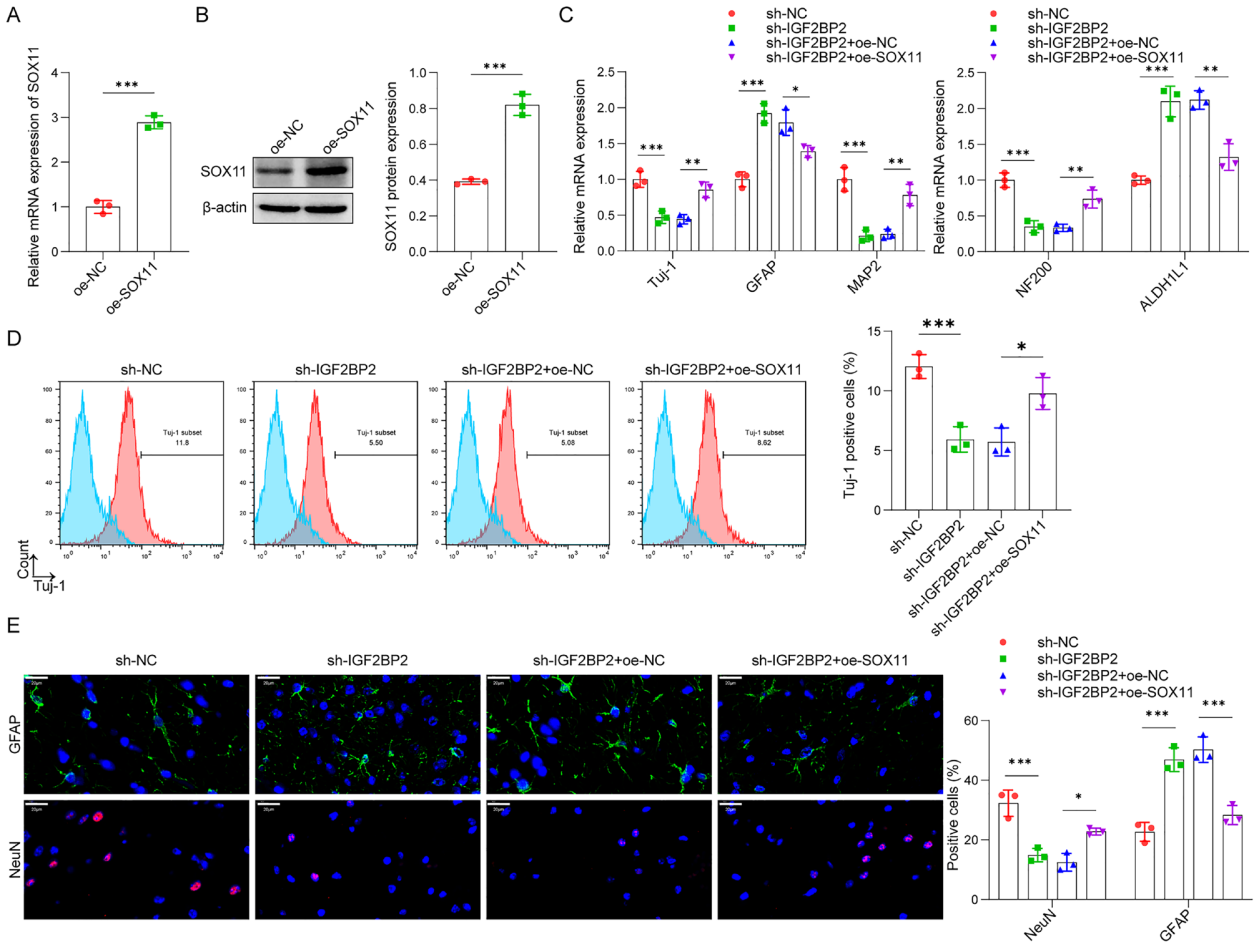
IGF2BP2 facilitated OM‐MSC neuronal differentiation through increasing SOX11. (A and B) qPCR and western blot analysis of SOX11 mRNA (oe‐NC: 1.00 ± 0.14, oe‐SOX11: 2.89 ± 0.15) and protein levels (oe‐NC: 0.39 ± 0.01, oe‐SOX11: 0.82 ± 0.06) in OM‐MSCs treated with oe‐NC or oe‐SOX11 for 48 h. (C) OM‐MSCs were infected with Ad and incubated with differentiation medium for four days. The mRNA levels of Tuj‐1 (sh‐NC: 1.00 ± 0.11, sh‐IGF2BP2: 0.47 ± 0.09, sh‐IGF2BP2 + oe‐NC: 0.44 ± 0.06, sh‐IGF2BP2 + oe‐SOX11: 0.85 ± 0.11), MAP2 (sh‐NC: 1.00 ± 0.16, sh‐IGF2BP2: 0.21 ± 0.08, sh‐IGF2BP2 + oe‐NC: 0.24 ± 0.07, sh‐IGF2BP2 + oe‐SOX11: 0.78 ± 0.15), NF200 (sh‐NC: 1.00 ± 0.10, sh‐IGF2BP2: 0.35 ± 0.08, sh‐IGF2BP2 + oe‐NC: 0.33 ± 0.05, sh‐IGF2BP2 + oe‐SOX11: 0.73 ± 0.13), GFAP (sh‐NC: 1.00 ± 0.11, sh‐IGF2BP2: 1.92 ± 0.14, sh‐IGF2BP2 + oe‐NC: 1.79 ± 0.18, sh‐IGF2BP2 + oe‐SOX11: 1.39 ± 0.08), and ALDH1L1 (sh‐NC: 1.00 ± 0.06, sh‐IGF2BP2: 2.10 ± 0.21, sh‐IGF2BP2 + oe‐NC: 2.12 ± 0.13, sh‐IGF2BP2 + oe‐SOX11: 1.32 ± 0.18) was measured by qPCR assay. (D) Flow cytometry assay was used to detect Tuj‐1 positive cells (sh‐NC: 12.05 ± 0.99, sh‐IGF2BP2: 5.92 ± 1.07, sh‐IGF2BP2 + oe‐NC: 5.71 ± 1.18, sh‐IGF2BP2 + oe‐SOX11: 9.78 ± 1.34). (E) Immunofluorescence was used to observe the number of GFAP (sh‐NC: 22.68 ± 3.19, sh‐IGF2BP2: 46.90 ± 3.96, sh‐IGF2BP2 + oe‐NC: 50.26 ± 4.25, sh‐IGF2BP2 + oe‐SOX11: 28.34 ± 3.20) and NeuN (sh‐NC: 32.35 ± 4.40, sh‐IGF2BP2: 14.91 ± 2.24, sh‐IGF2BP2 + oe‐NC: 12.52 ± 2.97, sh‐IGF2BP2 + oe‐SOX11: 22.82 ± 1.13)‐positive cells. Scale bar, 20 μm. *n* = 3. *p < 0.05, **p < 0.01, ***p < 0.001.

SOX11 overexpression resulted in elevated SOX11 expression levels (Figure 7A,B). To investigate whether IGF2BP2 facilitated OM‐MSC neuronal differentiation by increasing SOX11 levels, we conducted IGF2BP2 depletion in OM‐MSCs both alone and in conjunction with SOX11 addition. As anticipated, IGF2BP2 knockdown significantly decreased Tuj‐1, MAP2, and NF200 expression while increasing GFAP and ALDH1L1 levels. These effects were partially reversed by SOX11 overexpression (Figure 7C). Moreover, flow cytometry (Figure 7D) and immunofluorescence (Figure 7E) assays confirmed that IGF2BP2 depletion decreased Tuj‐1 and NeuN‐positive cells, while enhancing GFAP‐positive cells, an effect that was negated by the addition of SOX11. Therefore, we conclude that IGF2BP2 promoted OM‐MSC neuronal differentiation by regulating SOX11.

## 4 Discussion

ICH poses a significant public health threat, characterized by high mortality rates and poor patient outcomes [19]. Understanding the mechanisms underlying neural damage could pave the way for innovative and effective treatments after ICH. MSCs have been shown to differentiate into neuron‐like cells and promote neural stem cell proliferation, aiding in the repair of damaged nerve tissues [20]. Notably, our previous study revealed that OM‐MSC neuronal differentiation contributes to the reduction of ICH‐triggered nerve damage [12]. However, the underlying mechanisms by which OM‐MSC neuronal differentiation reduces ICH‐induced nerve damage require further investigation. In this study, IGF2BP2 mitigates ICH‐induced brain damage by accelerating OM‐MSC neuronal differentiation. Mechanistically, IGF2BP2 is modified by SUMO1 and deSUMOylated by SENP1. Moreover, IGF2BP2 interacts with SOX11 to enhance its expression. Our findings provide a new perspective on promoting OM‐MSC neuronal differentiation and mitigating ICH‐induced brain damage.

OM‐MSCs provide neuroprotection in both ischemic stroke and hemorrhagic conditions by regulating angiogenesis, inflammation, and oxidative stress, thereby preventing neuronal death (e.g., apoptosis and pyroptosis) and promoting neurological recovery [8, 21, 22]. Moreover, Huang et al. revealed that co‐culturing curcumin with OM‐MSCs in ICH models more effectively prevented neuronal death and brain tissue damage resulting from ferroptotic toxicity [18]. In light of the protective effect of OM‐MSCs on neuronal differentiation in ICH‐related brain injury, we aimed to explore the underlying mechanisms. It has been reported that the overexpression of IGF2BP2 enhances the neurogenic potential and inhibits astrocytic differentiation of neural precursor cells [16]. Here, we reveal that IGF2BP2 elevates the protective effect of OM‐MSCs against ICH‐induced brain injury. In addition, the protective effect of IGF2BP2 against ICH‐induced brain injury is mediated by promoting the neuronal differentiation of OM‐MSCs.

SUMOylation plays a crucial role in various cellular processes, including maintaining genome integrity, signal transduction, transcription, and nuclear transport, through essential protein modifications [23]. SUMOylation is catalyzed by SUMO‐specific activating, conjugating, and ligase enzymes [24]. SENP1, a SUMO‐specific protease, provides neuroprotection in I/R injury by inhibiting neuronal apoptosis [25]. The deSUMOylation of Sirt3 regulated by SENP1 is critical for protecting the brain from I/R injury [26]. Using the SUMOplot Analysis Program (https://www.abcepta.com/sumoplot), we identified high‐confidence SUMO modification sites in IGF2BP2, suggesting that IGF2BP2 may be regulated by SENP1‐mediated deSUMOylation. As anticipated, we confirmed that SENP1 triggers IGF2BP2 deSUMOylation via SUMO1. Consistent with the findings of a previous study [12], SENP1 promoted OM‐MSC neuronal differentiation. Furthermore, we observed that the depletion of IGF2BP2 partially reversed the promoting effect of SENP1 on neuronal differentiation in this study.

RBPs are proteins that specifically bind to RNAs within cells and are involved in various post‐transcriptional regulatory functions, including transport, translation, RNA splicing, and localization [27]. Consequently, RBPs can participate in mRNA formation, post‐transcriptional regulation, and translation through their interactions, thereby influencing mRNA functionality [28]. Through the ENCORI database, we identified IGF2BP2 as associated with SOX11 mRNA. Upregulation of SOX11 expression has been shown to enhance recovery from ischemic nerve injury [29]. Moreover, SOX11 is crucial for cell survival, regulating neuronal survival, and plays a pivotal role in sensory neuron development [30]. The upregulation of RNA‐binding motif protein 3 (RBM3) has been linked to increased neuronal differentiation of neural stem cells through the enhancement of SOX11 expression [31]. Therefore, we hypothesized that IGF2BP2 may influence OM‐MSC differentiation by regulating SOX11. As anticipated, IGF2BP2 was found to interact with SOX11, promoting its expression. This interaction facilitated the neuronal differentiation of OM‐MSCs via the upregulation of SOX11, thereby mitigating brain injury triggered by ICH. Despite the valuable insights provided by our study, several limitations must be acknowledged. First, our investigation primarily focused on the acute phase within 72 h post‐ICH, aiming to elucidate the role of IGF2BP2 deSUMOylation in promoting OM‐MSC‐mediated neuronal differentiation through SOX11 stabilization. This time frame was selected due to the biological significance of the early differentiation process and the clinical importance of the 72‐h window, during which maximal cerebral edema and neurological deficits typically stabilize. However, we recognize that the lack of long‐term outcome assessments limits our understanding of the sustained therapeutic effects and functional recovery. Future studies involving extended observation periods and behavioral evaluations will be essential to comprehensively evaluate the durability and translational potential of our findings.

Beyond the IGF2BP2/SOX11 axis identified in our study, other mechanisms may also play a role in regulating OM‐MSC neuronal differentiation and the repair of ICH‐induced brain injury. Previous studies have demonstrated that signaling pathways such as Wnt/β‐catenin, Notch, PI3K/AKT, and MAPK/ERK are crucial for stem cell differentiation and neural regeneration [32, 33, 34, 35]. It is plausible that these pathways may interact with or operate in parallel to the IGF2BP2/SOX11 axis. Furthermore, other post‐transcriptional regulators, including microRNAs and long non‐coding RNAs, have been reported to modulate neural differentiation by targeting RBPs or transcription factors. For example, miR‐34a [36] and miR‐124 [37] are well‐known for their roles in neurogenesis and could potentially influence OM‐MSC fate through direct or indirect interactions with IGF2BP2 or SOX11. Additionally, epigenetic modifications and metabolic reprogramming may also contribute to the observed phenotypic changes. Therefore, future studies are warranted to explore these alternative or complementary mechanisms in greater detail, which could lead to a more comprehensive understanding of OM‐MSC‐mediated neuroprotection in ICH.

In terms of clinical implications, our findings suggest that targeting the deSUMOylation of IGF2BP2 in OM‐MSCs to enhance SOX11 stability may offer a novel therapeutic approach for patients suffering from ICH. OM‐MSCs are readily accessible and exhibit low immunogenicity, making them promising candidates for autologous transplantation. By promoting neuronal differentiation, these modified OM‐MSCs may enhance tissue repair and functional recovery in ICH patients. Moreover, the IGF2BP2/SOX11 pathway may be further explored in pharmacological interventions aimed at boosting endogenous repair mechanisms post‐ICH. Future studies will be necessary to validate these findings in larger animal models and ultimately in clinical trials.

**FIGURE 8:**
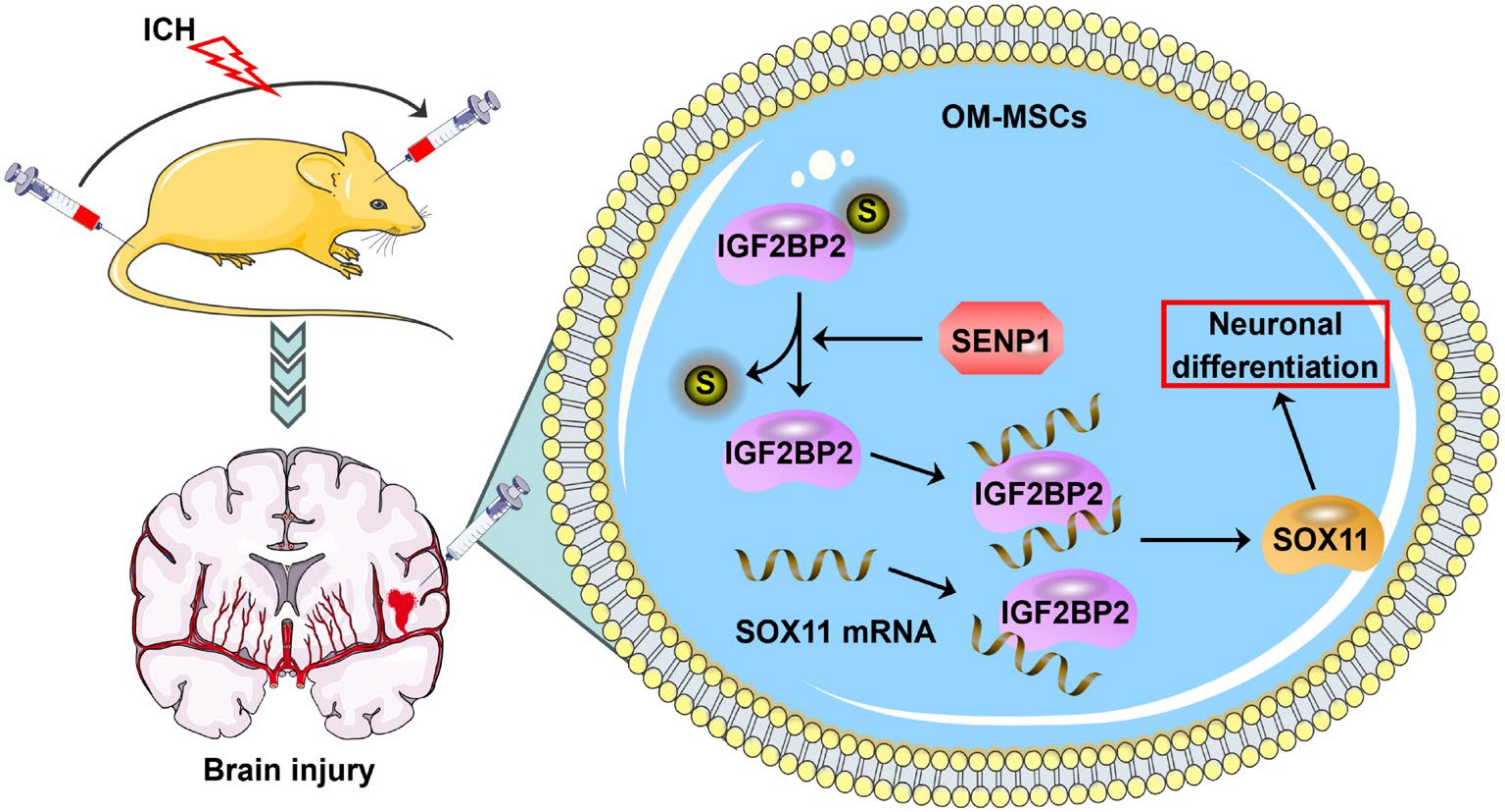
Schematic diagram of the mechanism in this study. The mechanism schematic diagram illustrates how the SENP1/IGF2BP2/SOX11 axis regulates neuronal differentiation in OM‐MSCs. It highlights the cascade of molecular interactions that promote neuronal differentiation and mitigate brain damage following ICH.

In conclusion (Figure 8), SENP1 enhances the binding of IGF2BP2 to SOX11 mRNA by mediating its deSUMOylation, thereby enhancing SOX11 mRNA stability and facilitating OM‐MSC neuronal differentiation. The SENP1/IGF2BP2/SOX11 axis plays a crucial role in protecting against ICH‐induced brain damage by promoting OM‐MSC neuronal differentiation. Our findings offer new insights into potential treatments for ICH‐induced brain injury.

## Author Contributions

Jun He, Yuhan Luo guaranteed the integrity of the entire study. Jun He, Yuhan Luo, Ying Xia designed the study and literature research. Chuang Wang, Chonghua Jiang defined the intellectual content. Chuang Wang, Chonghua Jiang performed the experiment. Jian Wang, Ying Xia collected the data. Jian Wang, Ying Xia analyzed the data. Jun He, Yuhan Luo, Ying Xia wrote the main manuscript and prepared figures. All authors reviewed the manuscript.

## Ethics Statement

This study was conducted under the guidance of the Ethics Committee of the Haikou Affiliated Hospital of Central South University Xiangya School of Medicine (ethics approval no. 2024–89).

## Consent

The authors have nothing to report.

## Conflicts of Interest

The authors declare no conflicts of interest.

## Data Availability

The datasets used or analyzed during the current study are available from the corresponding author on reasonable request.
